# Supplementary material for: F-Box/WD Repeat Domain-Containing 7 Induces Chemotherapy Resistance in Colorectal Cancer Stem Cells
Source: Cancers (Basel). 2019 May 7;11(5):635. doi: 10.3390/cancers11050635 (PMC6562509; doi:10.3390/cancers11050635)
Supplement: Supplementary file 1 [file cancers-11-00635-s001.zip › Figure S1.pptx]

## Slide 1
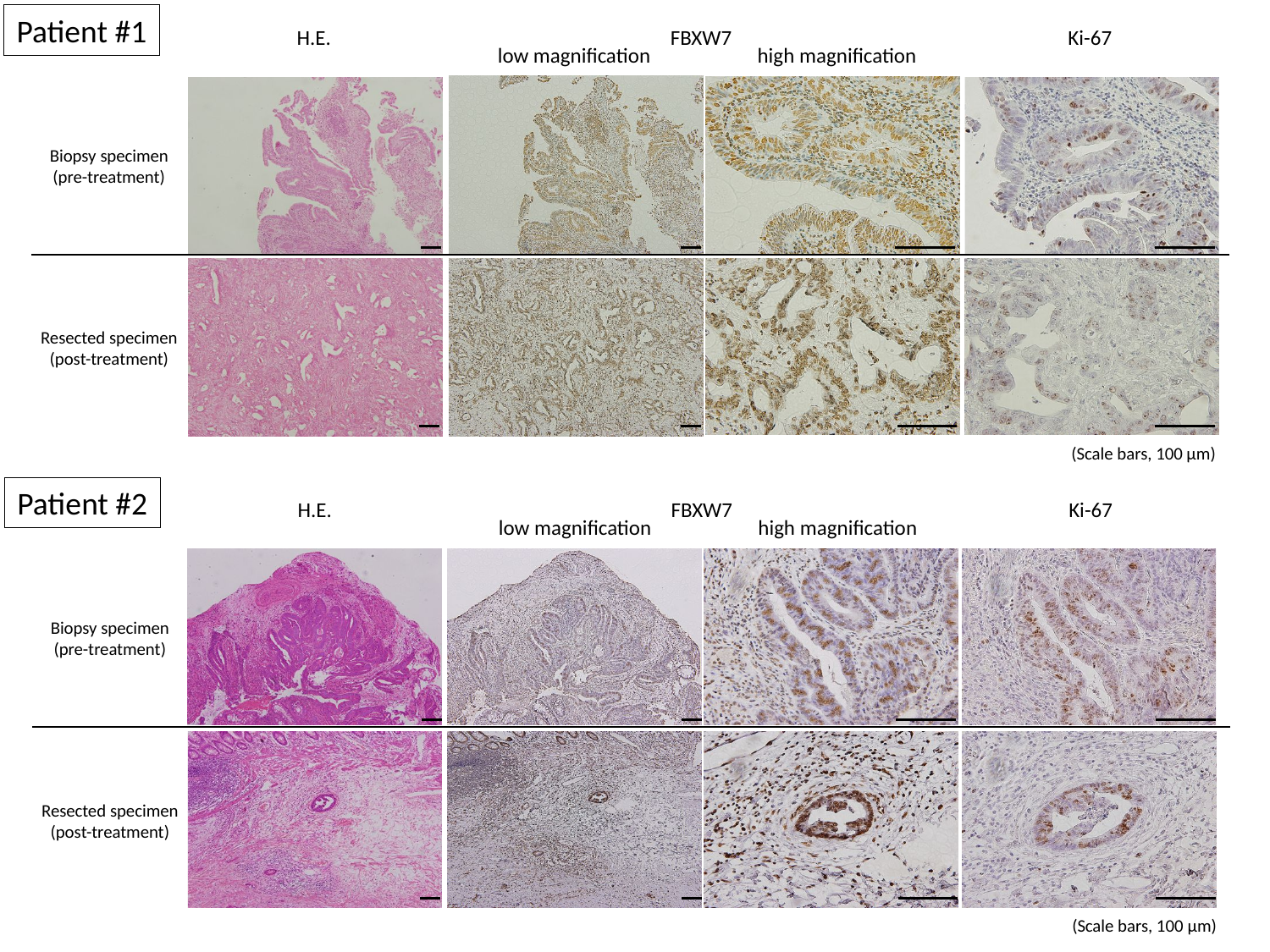

Patient #1
H.E.
FBXW7
Ki-67
low magnification
high magnification
Biopsy specimen
(pre-treatment)
Resected specimen
(post-treatment)
(Scale bars, 100 µm)
Patient #2
H.E.
FBXW7
Ki-67
low magnification
high magnification
Biopsy specimen
(pre-treatment)
Resected specimen
(post-treatment)
(Scale bars, 100 µm)

## Slide 2
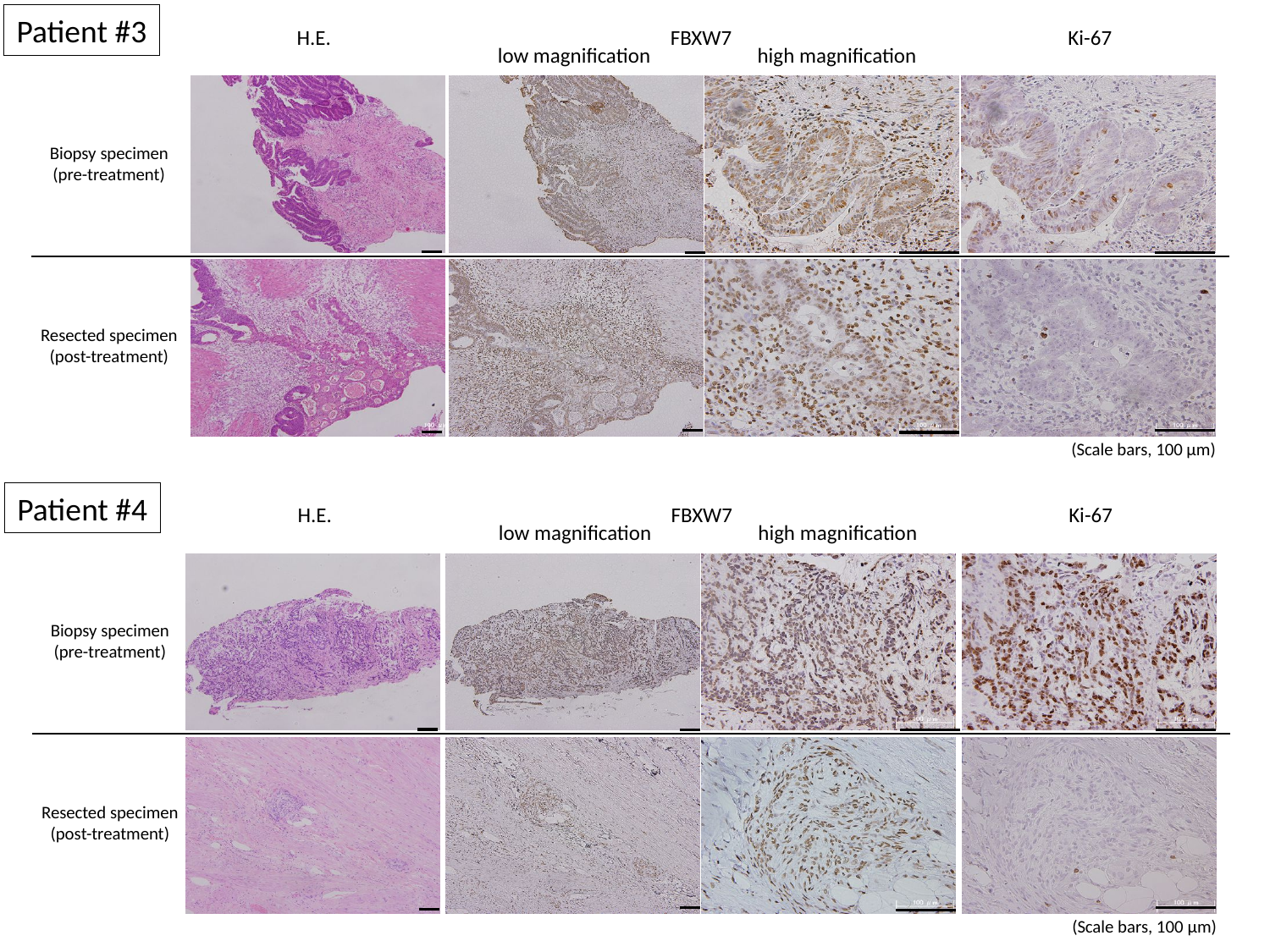

Patient #3
H.E.
FBXW7
Ki-67
low magnification
high magnification
Biopsy specimen
(pre-treatment)
Resected specimen
(post-treatment)
(Scale bars, 100 µm)
Patient #4
H.E.
FBXW7
Ki-67
low magnification
high magnification
Biopsy specimen
(pre-treatment)
Resected specimen
(post-treatment)
(Scale bars, 100 µm)
