# Supplementary material for: F-Box/WD Repeat Domain-Containing 7 Induces Chemotherapy Resistance in Colorectal Cancer Stem Cells
Source: Cancers (Basel). 2019 May 7;11(5):635. doi: 10.3390/cancers11050635 (PMC6562509; doi:10.3390/cancers11050635)
Supplement: Supplementary file 1 [file cancers-11-00635-s001.zip › Figure S2.pptx]

## Slide 1
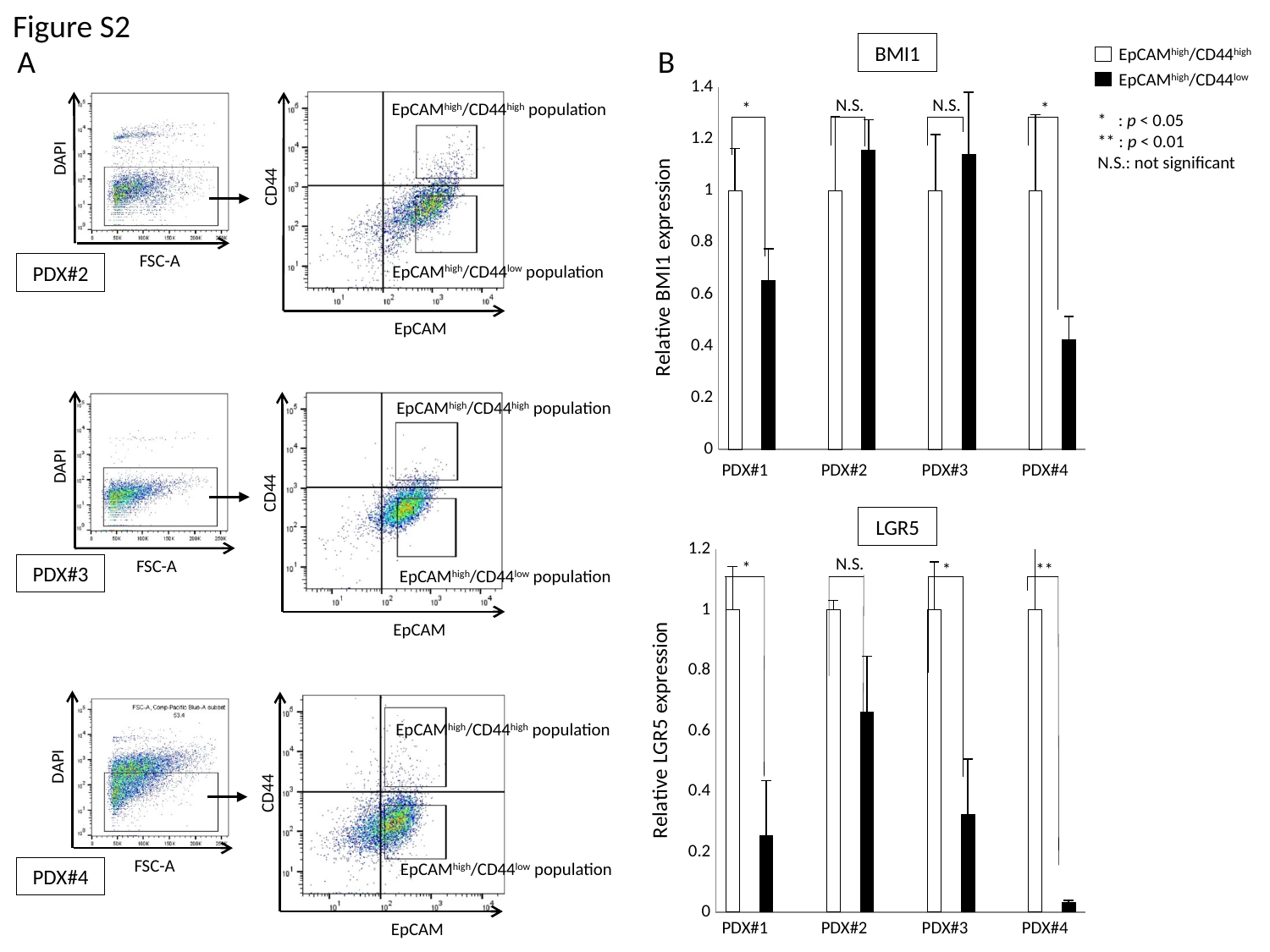

Figure S2
BMI1
A
B
EpCAMhigh/CD44high
EpCAMhigh/CD44low
### Chart
| Category | |
|---|---|
N.S.
N.S.
*
*
EpCAMhigh/CD44high population
* : p < 0.05
** : p < 0.01
N.S.: not significant
DAPI
CD44
FSC-A
EpCAMhigh/CD44low population
PDX#2
EpCAM
EpCAMhigh/CD44high population
DAPI
PDX#2
PDX#1
PDX#3
PDX#4
CD44
LGR5
### Chart
| Category | |
|---|---|N.S.
FSC-A
*
*
**
PDX#3
EpCAMhigh/CD44low population
EpCAM
EpCAMhigh/CD44high population
DAPI
CD44
FSC-A
EpCAMhigh/CD44low population
PDX#4
PDX#2
PDX#1
PDX#3
PDX#4
EpCAM
