# Supplementary material for: F-Box/WD Repeat Domain-Containing 7 Induces Chemotherapy Resistance in Colorectal Cancer Stem Cells
Source: Cancers (Basel). 2019 May 7;11(5):635. doi: 10.3390/cancers11050635 (PMC6562509; doi:10.3390/cancers11050635)
Supplement: Supplementary file 1 [file cancers-11-00635-s001.zip › Figure S3.pptx]

## Slide 1
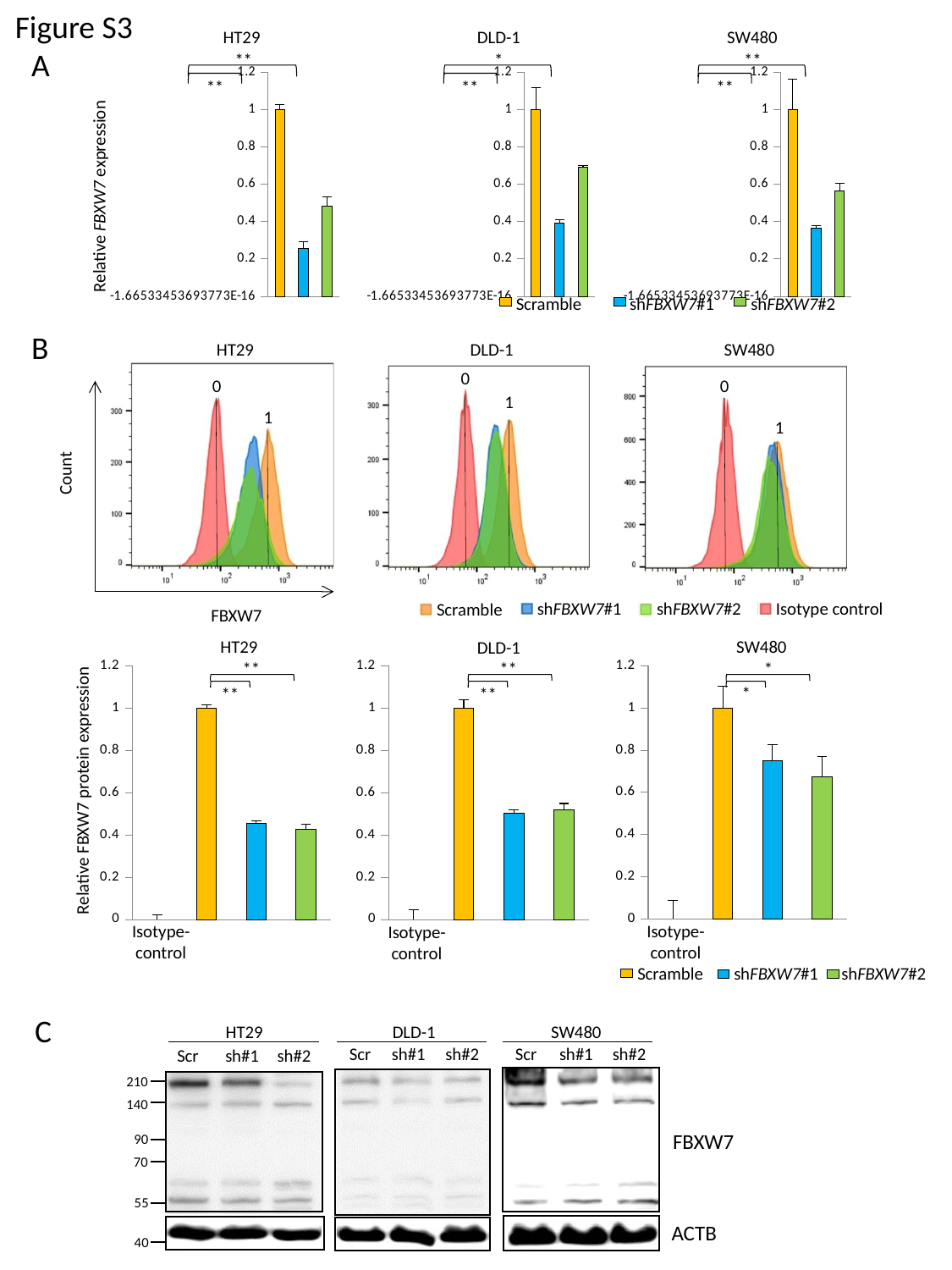

Figure S3
HT29
DLD-1
SW480
A
**
*
**
### Chart
| Category | |
|---|---|
| scr | 1.0 |
| #1 | 0.25466273694662767 |
| #2 | 0.482116158537191 |
### Chart
| Category | |
|---|---|
| scr | 1.0000000000000002 |
| #1 | 0.39357462110398783 |
| #2 | 0.691023160087718 |
### Chart
| Category | |
|---|---|
| scr | 1.0 |
| #1 | 0.36464252132333236 |
| #2 | 0.565476085642521 |**
**
**
Relative FBXW7 expression
Scramble
shFBXW7#1
shFBXW7#2
B
HT29
DLD-1
SW480
0
0
0
1
1
1
Count
shFBXW7#1
Isotype control
shFBXW7#2
Scramble
FBXW7
HT29
SW480
DLD-1
**
**
*
### Chart
| Category | |
|---|---|
| Isotype control | 0.0 |
| Scramble | 1.0 |
| shFBXW7#1 | 0.45309734513274336 |
| shFBXW7#2 | 0.42654867256637163 |
### Chart
| Category | |
|---|---|
| Isotype control | 0.0 |
| Scramble | 1.0 |
| shFBXW7#1 | 0.5041322314049587 |
| shFBXW7#2 | 0.5171192443919717 |
### Chart
| Category | |
|---|---|
| Isotype control | 0.0 |
| Scramble | 1.0 |
| shFBXW7#1 | 0.7490372272143774 |
| shFBXW7#2 | 0.6752246469833119 |
*
**
**
Relative FBXW7 protein expression
Isotype-control
Isotype-control
Isotype-control
Scramble
shFBXW7#1
shFBXW7#2
C
HT29
DLD-1
SW480
Scr
sh#1
sh#2
Scr
sh#1
sh#2
Scr
sh#1
sh#2
210
140
FBXW7
90
70
55
ACTB
40
