# Supplementary material for: F-Box/WD Repeat Domain-Containing 7 Induces Chemotherapy Resistance in Colorectal Cancer Stem Cells
Source: Cancers (Basel). 2019 May 7;11(5):635. doi: 10.3390/cancers11050635 (PMC6562509; doi:10.3390/cancers11050635)
Supplement: Supplementary file 1 [file cancers-11-00635-s001.zip › Figure S4.pptx]

## Slide 1
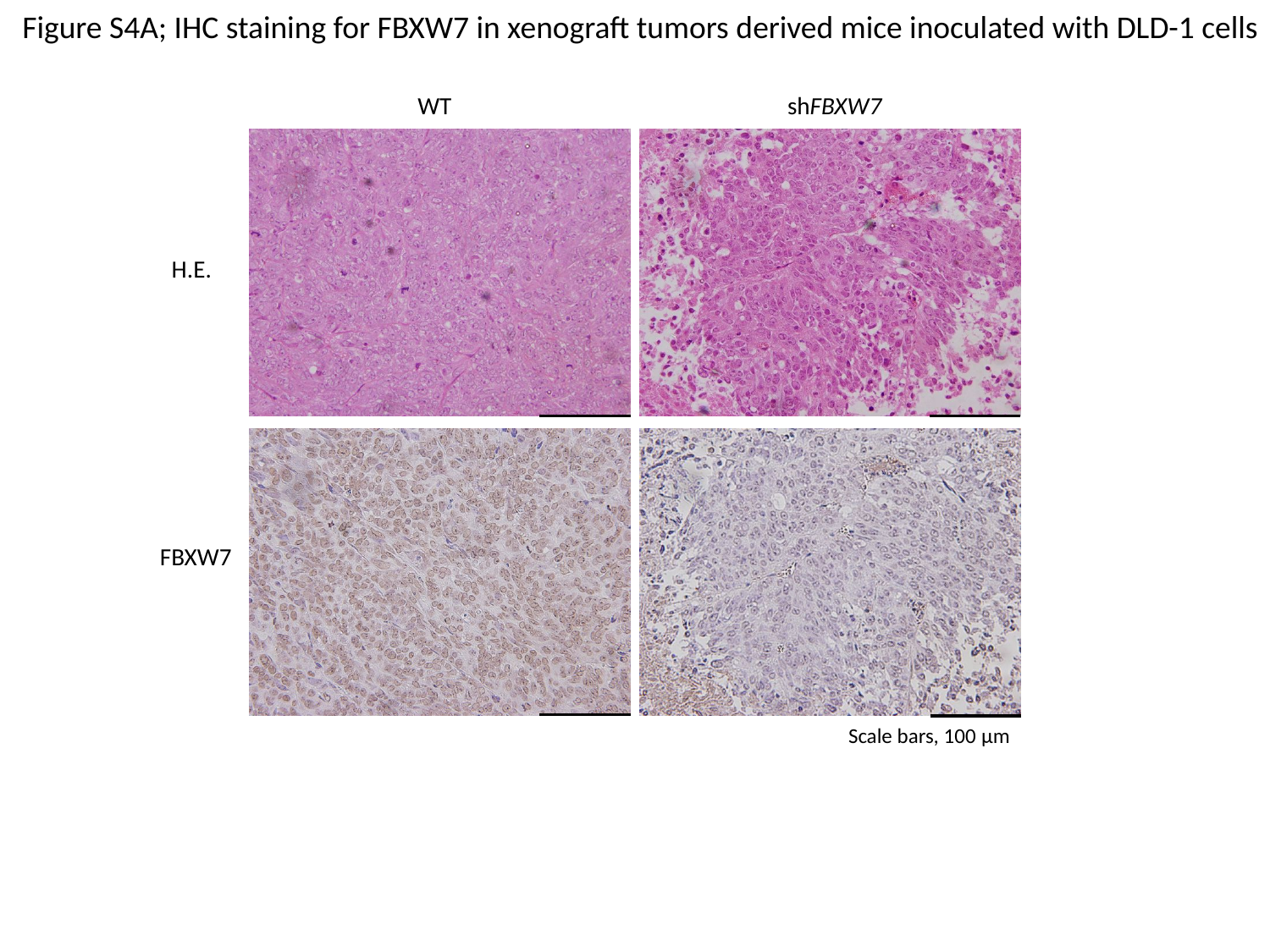

Figure S4A; IHC staining for FBXW7 in xenograft tumors derived mice inoculated with DLD-1 cells
WT
shFBXW7
H.E.
FBXW7
Scale bars, 100 μm

## Slide 2
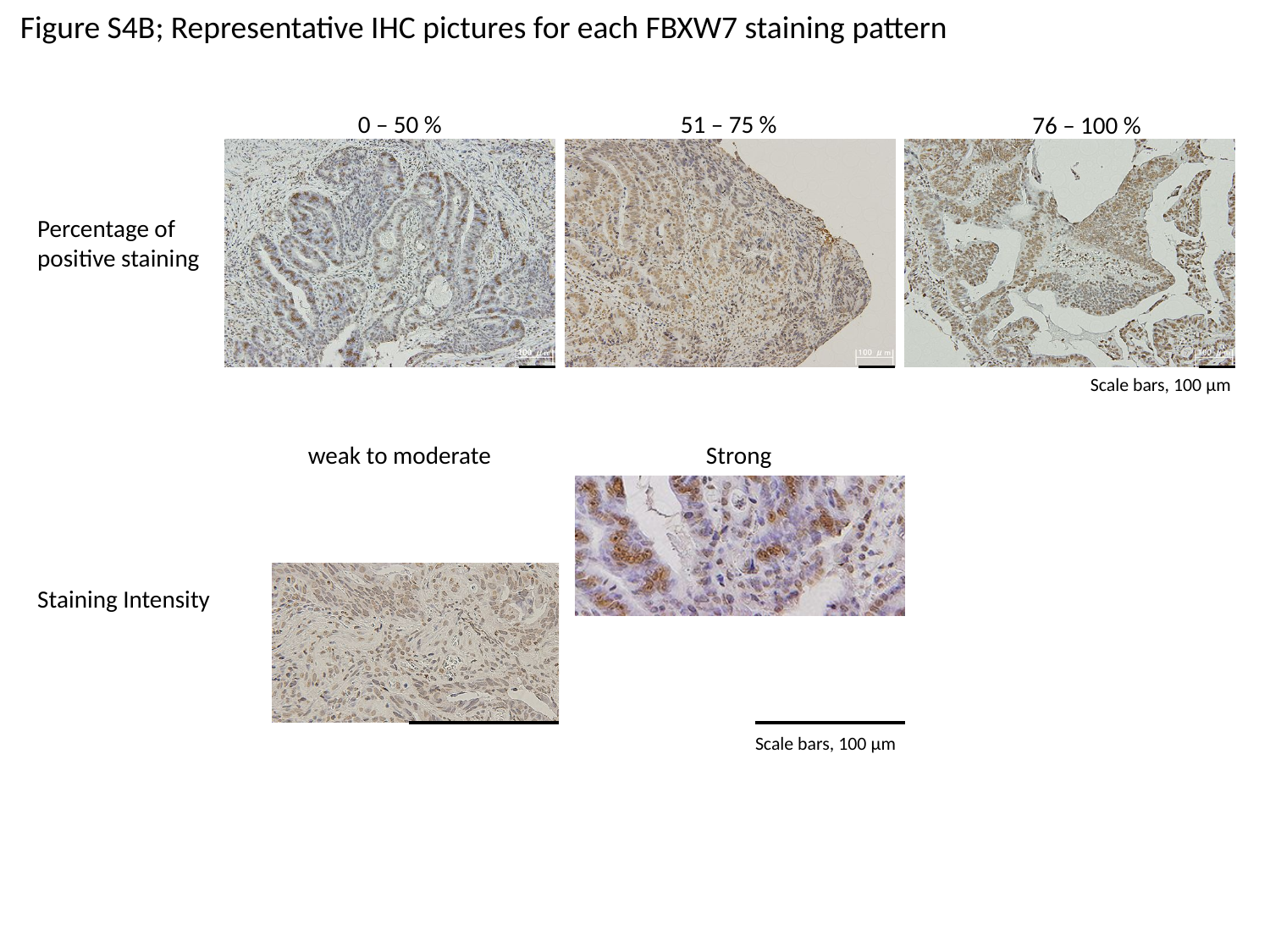

Figure S4B; Representative IHC pictures for each FBXW7 staining pattern
0 – 50 %
51 – 75 %
76 – 100 %
Percentage of
positive staining
Scale bars, 100 μm
weak to moderate
Strong
Staining Intensity
Scale bars, 100 μm
